# Developed community standards on setting the diffraction resolution yields improved density maps and a new monosaccharide model in pdb_00004dda

**DOI:** 10.1107/S2414314626008515

**Published:** 2026-08-28

**Authors:** John R. Helliwell, Robbie P. Joosten, Loes Kroon Batenburg, Simon Tanley

**Affiliations:** a Department of Chemistry, University of Manchester, Manchester, M13 0PL, United Kingdom; b Division of Biochemistry Netherlands Cancer Institute, 1066CX Amsterdam The Netherlands; c Utrecht University, 3584 CG Utrecht, The Netherlands; University of Southampton, United Kingdom

**Keywords:** raw diffraction data archiving, community data metrics, raw data reprocessing, electron-density model fitting

## Abstract

A systematic survey of monosaccharides in the PDB archive threw up a possible improvement in model fit in pdb_00004dda. Reprocessing of the publicly open archive of the raw diffraction images was made using *EVAL*. The diffraction resolution was assessed by the CC_1/2_ = 0.5 criterion and confirmed by paired model refinement implemented in *PDB-REDO*. The resulting change in diffraction resolution, from 2.4 Å to 2.0 Å, improved the clarity of the electron density at the monosaccharide binding site. The NAG (*N*-acetyl-β-d-glucosa­mine) was refitted as NDG (*N*-acetyl-α-d-glucosa­mine). This example illustrates the advantages of raw diffraction data archiving, as aimed at by FAIR data polices, allowing reprocessing as agreement on community metrics.

## Abstract 2

A query over the electron density fit of a monosaccharide in pdb_00004dda was resolved through applying the data metrics of the raw diffraction data processing that have evolved though the decade since the original publication. This improved the electron-density quality to allow a definitive model fit.

## Graphical abstract

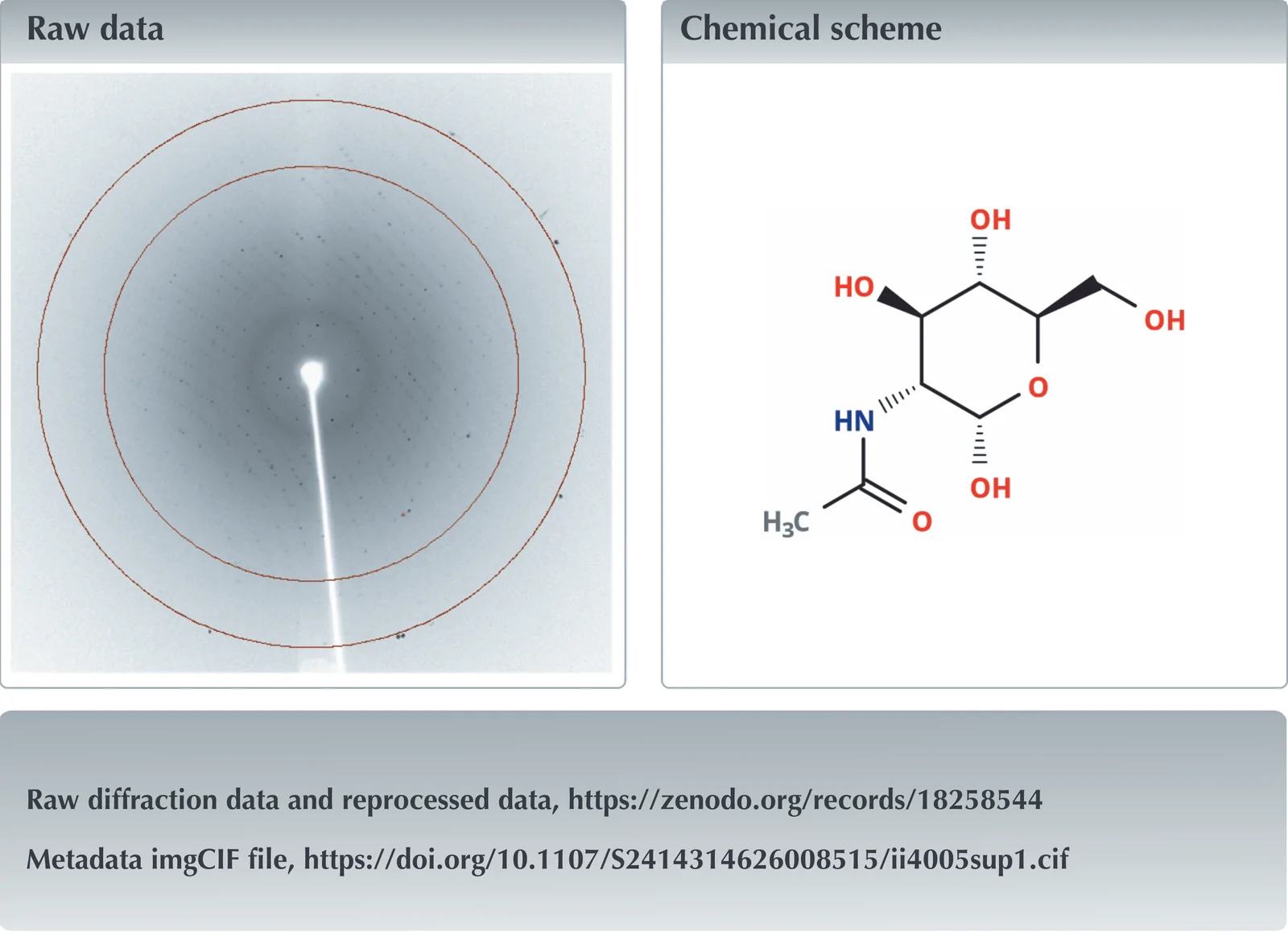

## Introduction

A systematic survey of monosaccharides in the PDB archive threw up a possible improvement in model fit in pdb_00004dda. The structure was published in Tanley *et al.* (2012 ▸) with a diffraction resolution of <*I*/σ*I*> = 4.0. The ways to establish the diffraction resolution have since then developed into community-agreed improved criteria. Reprocessing of the publicly open archive of the raw diffraction images was made using *EVAL* (Schreurs *et al.* 2010 ▸). The diffraction resolution was assessed by the CC_1/2_ = 0.5 criterion (Karplus & Diederichs 2012 ▸) and confirmed by paired-model refinement using *Refmacat* (Yamashita *et al.* 2023 ▸) implemented in *PDB-REDO* (Joosten *et al.* 2014 ▸). The resulting change in diffraction resolution, from 2.4 Å to 2.0 Å, improved the clarity of the electron density at the monosaccharide binding site. The NAG (*N*-acetyl-β-d-glucosa­mine) was refitted as NDG (*N*-acetyl-α-d-glucosa­mine). The raw diffraction images for pdb_00004dda in the original RAXIS.osc format, along with the reprocessing files, can be found at https://zenodo.org/records/18258544. The raw data were converted to fullCBF with the make-cbf tool as part of *imgCIF_Creator* (Kluyver *et al.*, 2026 ▸) and deposited to Zenodo (https://zenodo.org/records/20326720). This example illustrates the advantages of raw diffraction data archiving, as aimed at by FAIR data polices (Wilkinson *et al.*, 2016 ▸), allowing reprocessing as agreement on community metrics improve.

The benefits of raw diffraction data archiving linked to deposited PDB files were described with an example by the IUCr abstract (Helliwell *et al.*, 2023 ▸) as a case study within the Global Open Science Cloud (Chen *et al.* 2023 ▸). Their example documented a case of a good choice of diffraction limit by the authors (Sato *et al.* 2021 ▸) and PDB entry pdb_00007ccy. In the case of pdb_00004dda we can now illustrate below the corrective power and an improvement with one of our own studies.

## Methods and results

**Figure 1:**
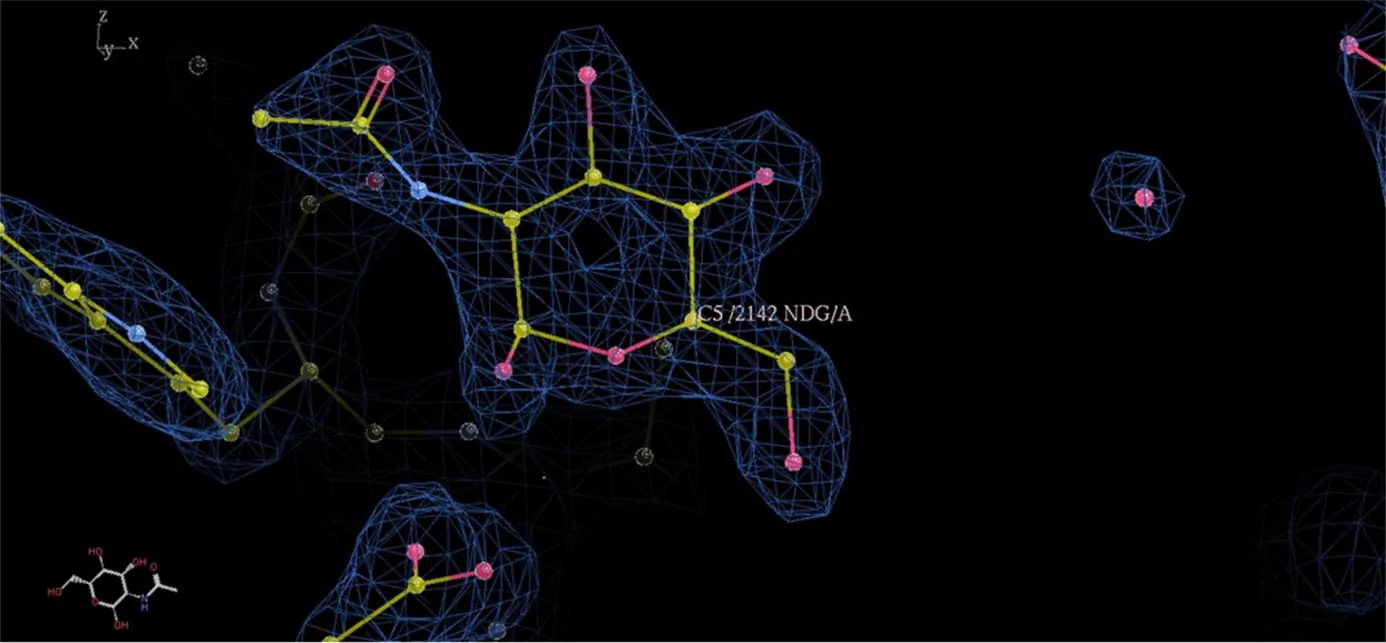
Electron density 2*mF*_o_−*DF*_c_ (1.58 r.m.s.) with the monosaccharide NDG fitted (this study at 2.0 Å). The real-space correlation coefficient of NDG is 0.93. This figure was produced using *Coot* (Emsley *et al.*, 2010 ▸).

Data were processed in the same way as in Tanley *et al.* (2012 ▸) and detailed in Tanley *et al.* (2013 ▸) by *EVAL* (Schreurs *et al.* 2010 ▸), which uses a diffraction data integration method based on *ab initio* predicted profiles, but only extending the resolution and with the summary statistics now including CC_1/2_. The new diffraction resolution was chosen to be at a CC_1/2_ criterion of 0.5 and confirmed by paired model refinement implemented in *PDB-REDO* (Joosten *et al.* 2014 ▸). The electron density at the monosaccharide binding site after the reprocessing and re-refinement is shown in Fig. 1 ▸. The raw data collection details are provided in Table 1 ▸. The data statistics of pdb_00004dda and of the reprocessed and re-refined models are given in Table 2 ▸. The metadata for the raw diffraction images have been automatically checked by the IUCrData checkcif-for-raw-data software (https://publbio.iucr.org/imgcif/).

## Discussion and conclusions

As community metrics in macromolecular crystallography evolve, the processing of archived raw diffraction images can be successfully re-evaluated as shown in this case with an improved diffraction resolution limit and model. The full range of situations suitable for a *Raw Data Letter* are described in Kroon-Batenburg *et al.* (2022 ▸).

## Supplementary Material

Metadata imgCIF file. DOI: 10.1107/S2414314626008515/ii4005img.cif

CheckCIF for raw data report. DOI: 10.1107/S2414314626008515/ii4005img_check.pdf

mtz file. DOI: 10.1107/S2414314626008515/ii4005sup1.txt

mmCIF file for structure 2WIY. DOI: 10.1107/S2414314626008515/ii4005sup2.txt

## Figures and Tables

**Table 1:** Experimental details

Raw Data	
DOI	https://doi.org/10.5281/zenodo.18258544
	https://doi.org/10.5281/zenodo.20326720
Data archive	Zenodo
Data Format	OSC and CBF
	
Data Collection	
Diffractometer	Rigaku Micromax-007 generator
Detector type	R-AXIS IV image plate
Radiation type	Cu *K*α
Wavelength (Å)	1.5418
Beam centre (mm) (fast, slow)	150.00, −149.89
Detector axis	−*Z*
Detector distance (mm)	140.00†
Pixel size (mm)	0.100 × 0.100
No. of pixels	3000 × 3000
No. of scans	1
Scan axis	ω, χ
Start angle, increment per frame (°)	0.0, 1.0
Scan range (°)	180.0
No. of frames	180
Exposure time per frame (s)	4.5

The detector distance in the raw image files is given as 140.0 mm, while after indexing and refinement it is approximately 135 mm.

**Table 2:** 4dda X-ray crystallographic data processed via *EVAL15* and refinement statistics for HEWL co-crystallized with *N*-acetyl-D-glucosamine (Tanley *et al* 2012 ▸) and new data processing and refinement statistics undertaken by *PDB-REDO* with TLS refinements and hydrogens in riding positions

Space group	*P*4_3_2_1_2	*P*4_3_2_1_2
Unit-cell parameters (Å)	*a* = *b* = 78.37, *c* = 36.58	*a* = *b* = 78.37, *c* = 36.58
Molecular mass (Da)	14700	14700
Molecules per asymmetric unit	1	1
Observed reflections	49543	93073 (10160)
Unique reflections	4120	7468 (796)
Resolution (Å)	19.59–2.40 (2.48–2.40)	19.59–2.0 (2.07–2.0)
Completeness (%)	85.3 (100)	91.5 (100.0)
*R*_merge_	0.147 (0.607)	0.243 (1.960)
<*I*/σ(*I*)>	13.6 (4.02)	8.15 (1.14)
CC_1/2_	†	0.996 (0.508)
Multiplicity	12.1 (12.8)	12.5 (12.8)
Cruickshank DPI (Å)	0.806	0.287
Average Bfactor (Å^2^)	29.5	21.9
Refinement *R*_factor_/*R*_free_(%)	20.0/28.5	20.4/26.0
R.m.s. *Z* bonds	1.201	0.204
R.m.s. *Z* angles	0.967	0.439
Molprobity Clashscore	5.53	2.00
Ramachandran *Z*-score	−2.12	−0.06
Rotamer normality Z-score	−1.88	−1.11

Note 1. CC_1/2_ was not a metric in use in 2012 ▸.

## Data Availability

The raw diffraction data and the reprocessed data to 2.0 Å using *EVAL* as well as the mol­ecular model are available at https://zenodo.org/records/18258544. The raw diffraction images converted to fullCBF format are available at https://zenodo.org/records/20326720. The improved Protein Data Bank entry can be found at pdb_000030IG. The pdb_00004dda entry is now obsolete.
