# Supplementary material for: Developed community standards on setting the diffraction resolution yields improved density maps and a new monosaccharide model in pdb_00004dda
Source: IUCrdata. 2026 Aug 28;11(Pt 8):x260851. doi: 10.1107/S2414314626008515 (PMC13548370; doi:10.1107/S2414314626008515)
Supplement: Supplementary file 1 [file x-11-x260851-img_check.pdf]

checkImgCIF report

Powered by https://github.com/jamesrhester/ImgCIFHandler.jl

```
 ImgCIF checker version 2023-03-14

Checking CIF block 4DDA_files

Running checks (no image download)
========================================


Testing: Required items: PASS

Testing: Data source: PASS

Testing: Axes defined: PASS

Testing: Our limitations: PASS

Testing: Detector translation: PASS

Testing: Scan range: PASS
   Range/increment match number of frames 180.0 for scan SCAN.1 (expected 180.0)

Testing: All frames present: PASS
   All frames present and correct for SCAN.1

Testing: Detector surface axes used properly: PASS

Testing: Pixel size and origin described correctly: PASS

Testing: Check calculated beam centre: PASS

Testing: Check principal axis is aligned with X: PASS
Testing presence of archive:

Testing: All archives are accessible: PASS

====End of Checks====
```
